# Patient Experience with Continuous Glucose Monitoring During Dialysis in Type 2 Diabetes: A Qualitative Study

**DOI:** 10.3390/jcm14196943

**Published:** 2025-09-30

**Authors:** Miguel Angel Cuevas-Budhart, Dante Atzin Juncos Ríos, Maricruz Ponce Villavicencio, Marcela Ávila Diaz, María Begoña Ilabaca Avendaño, Maricela Beatriz Rocha-Carrillo, Ramón Paniagua

**Affiliations:** 1 Unidad de Investigación Médica en Enfermedades Nefrológicas, Instituto Mexicano del Seguro Social, Mexico City 06720, Mexico; cramav@gmail.com; 2 School of Nursing, Faculty of Higher Studies Iztacala (FES Iztacala), Universidad Nacional Autónoma de México, Tlalnepantla 54090, Mexico; dantejuncos@gmail.com; 3 Hospital de Especialidades Centro Médico Nacional Siglo XXI, Mexico City 06720, Mexico; mponce3623@gmail.com; 4 Hospital General de Zona 1–A, Mexico City 03303, Mexico; jauregui0526@att.net.mx; 5 Hospital General Regional N°1 Mc Gregor, Mexico City 03103, Mexico; mariabrocarr@gmail.com

**Keywords:** continuous glucose monitoring, dialysis, diabetes management, patient experience, qualitative research, blood glucose control, chronic kidney disease

## Abstract

**Objective:** To explore the lived experiences of type 2 diabetes mellitus (T2DM) patients undergoing peritoneal dialysis (PD) or hemodialysis (HD) using continuous glucose monitoring (CGM). **Research Design and Methods**: A qualitative phenomenological study was conducted with 50 adult T2DM patients on PD or HD who used CGM for at least 14 days. Semi-structured interviews were audio-recorded and transcribed verbatim. A thematic analysis framework was applied to identify major themes regarding insulin management, CGM utilization, and emotional and social dimensions. **Results:** Four main themes emerged, each with multiple subthemes. PD patients emphasized enhanced autonomy and frequent insulin adjustments due to dialysate glucose absorption. Conversely, HD patients reported severe post-dialysis fatigue, emotional distress, and limited social engagement often associated with intra-dialytic hypoglycemia. CGM was valued by 85% of participants for improving metabolic awareness and self-management. However, 15% reported barriers such as device cost and technical difficulties. The insights clearly distinguish the differential impact of dialysis modality on daily glucose control and patient well-being. **Conclusions:** These findings underscore the critical need for patient-centered care incorporating access to CGM and tailored insulin regimens. Equitable implementation of CGM in dialysis settings could significantly enhance glycemic control, emotional resilience, and overall quality of life.

## 1. Introduction

The clinical management of patients with type 2 diabetes mellitus (T2DM) who are undergoing renal replacement therapy with peritoneal dialysis (PD) or hemodialysis (HD) poses a significant challenge. The coexistence of these two conditions complicates metabolic control. It limits the reliability of traditional markers such as glycated hemoglobin (HbA1c), which tends to underestimate actual glucose levels due to the reduced erythrocyte lifespan in advanced stages of Chronic Kidney Disease (CKD) [1,2].

In patients treated with PD, factors such as glucose absorption from dialysate solutions, the type and route of insulin administration, and dietary fluctuations require the use of more accurate technologies for glycemic monitoring [3,4,5].

In HD, the challenges are distinct. Significant glucose loss occurs during treatment sessions due to the permeability of dialysis membranes. These characteristics may contribute to glycemic imbalances both during and following dialysis. When the time of the HD session schedule or displacements to or from the HD facility and home occur at the usual eating time for the patients, they avoid the meal or reduce the ration size and do not constantly adjust insulin doses. At the same time, the physical exhaustion that often follows sessions, coupled with social limitations, frames a patient experience largely shaped by emotional and psychosocial factors rather than technical ones [6].

Continuous glucose monitoring (CGM) has opened new avenues for optimizing metabolic control and enhancing quality of life [7,8,9]. Nevertheless, the impact of these strategies from the patient’s perspective remains insufficiently explored.

Several studies have documented the perceived benefits of CGM, including reduced fear of hypoglycemia, a heightened sense of autonomy, and an improved perception of overall well-being [4,10,11]. However, reports have also noted increased anxiety associated with continuous device use, particularly among HD patients [8,10,11,12].

The implementation of CGM in dialysis settings faces both technical and economic barriers. Issues such as false alarms, connectivity problems, frequent calibration requirements, and high costs hinder sustainable adoption by Ling et al., 2022 [7]. While the clinical efficacy of CGM has been well established in non-renal patients with Diabetes, a critical gap remains regarding the subjective experiences of patients with renal disease. Prior qualitative studies [13], Gerogianni et al., 2019 [14], and Varela et al., 2011 [15] suggest that CGM use influences not only self-care practices but also bodily awareness and social isolation factors that are essential to understanding therapeutic adherence and the broader impact of treatment.

Against this backdrop, the present study was designed to explore and compare the lived experiences of patients with T2DM undergoing peritoneal or hemodialysis, focusing on insulin management, the use of CGM, and the emotional, physical, and social consequences of treatment.

## 2. Materials and Methods

### 2.1. Study Design

This study used a qualitative, exploratory approach grounded in the phenomenological paradigm [16,17]. This methodological orientation enabled an in-depth exploration of patients’ subjective experiences, particularly emphasizing the lived experience of using CGM as a metabolic control tool and the emotional, physical, and social impact of renal replacement therapy. In line with this approach, five semi-structured questions were crafted and presented to patients who consented to participate. These questions aimed to elicit personal narratives and explore key aspects of daily life while delving into the physiological, social, and psychological dimensions related to the challenges of diabetes amid its major complications and CKD. The interview questions were as follows:

1. What has your experience with diabetes been, and how has it affected your daily life?
2. What adjustments have you made in your daily lifestyle to manage your condition?
3. What has been your experience with dialysis therapy?
4. How have you felt using the continuous glucose monitoring device?
5. What is your opinion regarding CGM as a complementary tool for glucose control?

### 2.2. Participant Selection and Context

Participants were selected through purposive sampling, aiming to include individuals with direct experience of the phenomenon under investigation. Fifty patients with type 2 diabetes mellitus and end-stage kidney disease secondary to diabetic nephropathy, all of whom had previously participated in a research protocol and were undergoing either peritoneal dialysis or hemodialysis, were invited to take part in the study. Eligibility requires a minimum of 14 days of continuous glucose monitoring use.

Patients who were unable to provide informed consent, presented with acute intercurrent illness, had used CGM for fewer than 14 days, or had cognitive impairment that could compromise the reliability of their narratives were excluded. Prior exposure to CGM was established as an inclusion criterion, as the aim of the study was to explore the lived experiences of patients who had used this technology. Recruitment was conducted In Mexico City, Mexico, five hospitals were selected for their diversity in level of care and geographic location, enabling the inclusion of participants from various clinical settings and sociocultural backgrounds. The participating hospitals included Hospital de Especialidades, Centro Médico Nacional Siglo XXI (IMSS); Hospital General de Zona 1-A, Ciudad de México; Hospital General Regional N°1 Mc Gregor, Ciudad de México; and Hospital General de Zona with Family Medicine Unit No. 8 (IMSS), Mexico City, Mexico. The study was conducted between November 2024 and June 2025, with a minimum of 14 days of CGM use required for eligibility.

Theoretical saturation was reached when no new conceptual dimensions emerged from the interview analysis, with recurring patterns solidified around CGM use, insulin therapy, and the emotional and social impact of renal replacement therapy [17].

Patients from both therapies were included to compare experiences across dialysis modalities, covering technical and experiential aspects. The final sample size was determined by data saturation, ensuring adequate representation of clinical and experiential diversity, an essential requirement in phenomenological research.

Participants were recruited from HD, Continuous Ambulatory Peritoneal Dialysis (CAPD), and Automated Peritoneal Dialysis (APD) programs. While national data indicate that HD is currently more prevalent than PD in Mexico, our sample intentionally emphasized APD and CAPD patients to capture patient experiences in peritoneal dialysis. Including HD participants allowed a comparative perspective between modalities.

### 2.3. Data Collection

Interviews were conducted individually by telephone, which ensured multicenter feasibility across the five participating hospitals and facilitated the inclusion of patients facing socioeconomic barriers to travel, while allowing greater logistical flexibility and maintaining narrative depth. Each interview was guided by a semi-structured script designed to delve into personal experiences with CGM, its impact on self-care behaviors, and perceived changes in quality of life. Although non-verbal cues could not be captured, phenomenological techniques such as rephrasing, probing, and participant validation of responses were systematically applied to preserve the depth and fidelity of participants’ narratives, ensuring that perspectives were accurately expressed regardless of educational background.

### 2.4. Data Analysis

Interview transcripts were analyzed using an interpretative phenomenological framework. All interviews were transcribed verbatim and examined using MAXQDA software (version 22) for systematic analysis. An inductive coding strategy was applied, beginning with line-by-line coding to capture meaningful units of text. Both manual coding and AI-assisted semantic search modules facilitated the identification of recurrent patterns.

AI-assisted features were used exclusively to support the semantic search and retrieval of text segments, facilitating the preliminary organization of data. The interpretive work, including inductive coding, category development, and refinement of themes, was performed by the research team through iterative discussions, consensus, and participant validation. This ensured that the software functioned as a responsible complement rather than a substitute for in-depth human phenomenological analysis.

Codes were then iteratively grouped into broader categories through constant comparison and team discussions, which were subsequently refined into themes and subthemes in line with phenomenological principles. To enhance the rigor of the process, triangulation among researchers was conducted, consensus was reached during analytic meetings, and selected participants were contacted to validate the accuracy of the emergent categories. The analysis focused on themes related to insulin management, CGM use, and the emotional, physical, and social impact of renal replacement therapy.

## 3. Results

The study sample included 50 patients with T2DM undergoing either PD or HD. Twenty-eight were on PD (18 on continuous ambulatory peritoneal dialysis [CAPD] and 10 on automated peritoneal dialysis [APD]), and 22 were on HD. Of the participants, 46% were women, with a mean age of 59 years and an average body mass index (BMI) of 27.12 (Table 1).

The phenomenological analysis allowed the findings to be organized into four principal thematic axes: (1) glucose management in peritoneal dialysis (PD), (2) lifestyle changes during dialysis, (3) treatment experiences in hemodialysis (HD), and (4) reflections on CGM as a complementary strategy. Each axis comprises three subthemes that explore the challenges and meanings patients attributed to treatment and self-care (Table 2).

### 3.1. Glucose Management in PD Patients

Among patients with T2DM on PD, glycemic control was reported to be influenced by glucose absorption and the limitations of conventional insulin therapy. The use of CGM facilitated a greater understanding of glucose fluctuations and supported adjustments in self-care routines. Findings were categorized into three aspects: insulin use, impact of CGM, and perception of metabolic control.

#### 3.1.1. Insulin Use in PD

Managing insulin therapy in PD posed significant challenges, as patients faced unpredictable glycemic variability due to peritoneal glucose absorption. Narratives revealed uncertainty and difficulty adjusting insulin doses, affecting patients’ perceived control over their condition and generating insecurity in self-management:

*“I’m using insulin, but sometimes I feel like I can’t get it right; some days my glucose spikes even though I’m doing everything the same, and I don’t know if it’s the food or the dialysis.”*

*—CM4*

*“Yes, I use insulin, but I never really know when it’s going to hit harder (…) sometimes I take the same dose as usual and end up with low sugar.”*

*—HF2*

These testimonies underscored the connection between metabolic instability, neuromuscular impairments, and visual complications. From this perspective, patients’ narratives conveyed vulnerability and fear, particularly concerning proper insulin administration.

#### 3.1.2. Impact of CGM

The integration of CGM in PD patients improved their awareness of glucose fluctuations. Participants highlighted how continuous access to glycemic information changed their sense of control and enabled informed adjustments in self-care, promoting greater adherence and autonomy:

*“Using the monitor was a good experience because I didn’t have to check constantly whenever I needed to, I could just look at the device.”*

*—CM10*

*“It helped me see how the device worked in my body over those two weeks (…) it made me more aware (…).”*

*—CF2*

#### 3.1.3. Perception of Safety and Metabolic Control

Patients using CGM reported a heightened sense of security, enabling them to observe the immediate effects of dietary and therapeutic decisions. This clarity strengthened their sense of ownership and confidence in daily glucose self-management:

*“That’s when you really notice, it makes you conscious (…) I realized I can eat well, and it won’t spike my sugar.”*

*—HF2*

*“(…) I could see that when I ate certain foods, my sugar would spike, but when I ate lighter, it stayed stable. That gave me more confidence.”*

*—CF2*

### 3.2. Lifestyle Changes and Adaptation in PD

Living with T2DM while on PD requires a profound restructuring of daily life. Beyond glycemic control, patients described changes affecting diet, social interaction, daily routines, and autonomy. Adaptation demanded continuous adjustments, often relying on close family support to sustain long-term self-care.

#### 3.2.1. Adjustments in Diet and Physical Activity

Initiating PD requires changes in diet and physical activity, not only to manage diabetes but also to meet the demands of dialysis therapy. These adjustments went beyond behavior, representing an emotional journey of letting go of past practices, with implications for identity and well-being:

*“With the diet, basically, I’ve realized that if you eat in a balanced way, diabetes doesn’t cause problems (…).”*

*—HF2*

*“I can’t do the things I used to—can’t eat what I used to, can’t go out like before, a lot of things change (…).”*

*—CM7*

#### 3.2.2. Transformation in Social an Emotional Life

Both disease and treatment exerted a significant impact on patients’ social and emotional lives. Activities once enjoyed, working, traveling, and socializing became limited, leading to feelings of loss and isolation. This transformation was not only physical but symbolic, as patients redefined their roles within family, work, and community:

*“I’ve had the disease for 26 years, and yes, it’s changed everything. I used to work and be independent, but now I rely on my daughter for many things. I don’t go out much, just to the hospital or for walks with her.”*

*—CF1*

*“Living with diabetes hasn’t been easy. I don’t do what I used to. My routines changed. I used to go out, and I worked. Now everything revolves around meals, medications, and doctor’s visits. It’s not the same anymore.”*

*—AM1*

#### 3.2.3. Family Support as a Key Element of Self-Care

Family involvement emerged as a crucial factor for treatment adherence and emotional stability among patients with PD. Beyond logistical assistance with diet, medication management, and clinic visits, emotional support played a significant role in sustaining motivation for daily self-care. The active presence of family was perceived as alleviating the emotional burden associated with living with diabetes and undergoing PD:

*“My son is the one who takes care of everything—my diet, everything.”*

*—CM12*

*“He’s our caregiver, my youngest son (…). I’m with my family, they’re with me, my grandchildren visit, we spend time together—just like a family, you know? So, I try to keep the best attitude.”*

*—HF1*

### 3.3. HD Treatment Experience

For many participants, HD represented a profoundly different experience compared to PD, not only due to its technical characteristics but also due to its physical, emotional, and daily life implications. The rigidity of treatment schedules, post-session exhaustion, and social restrictions imposed additional challenges, requiring adaptation, redefinition of roles, and, in many cases, acceptance of new ways of living.

#### 3.3.1. Physical and Emotional Impact of Hemodialysis

Narratives described HD as a physically demanding treatment that compromised patients’ vitality. Post-dialysis fatigue, intense thirst, and a sense of dependency on the hospital emerged as central experiences, impacting not only the body but also perceptions of autonomy and control:

*“I’m trying to balance both hemodialysis and daily work because I’ve gone back to work. (…) On dialysis days, I leave exhausted, hungry, and very thirsty. But, well… it’s part of the treatment.”*

*—HF2*

*“After every dialysis session, I feel like my body betrays me. I get up from the machine and can barely walk, as if the weight of the world is on me. The fatigue is not just physical, it’s mental. The thirst drives me crazy because I know I can’t drink as much as I’d like (…)”*

*—HM1*

#### 3.3.2. Impact on Work and Daily Life

HD significantly interfered with patients’ ability to work and maintain autonomy. The necessity of adhering to a rigid hospital schedule limited the possibility of holding formal employment and affected social participation, forcing a reorganization of life priorities:

*“You just can’t do the same things anymore (…) you can’t eat, you can’t lift things—and as a man, that’s really hard to accept.”*

*—HM3*

*“After starting hemodialysis, I had to stop working. I leave the sessions very tired, and everything now revolves around the treatment. I’ve had to reorganize my life to fit the schedule.”*

*—HF2*

#### 3.3.3. Subjective Comparisons Between HD and PD

Patients who had experienced both dialysis modalities, or who compared their own situation with that of family members on HD, described emotionally charged contrasts. Automated Peritoneal Dialysis (APD) was perceived as offering greater flexibility and relative preservation of daily life, whereas HD was seen as limiting, exhausting, and marked by dependency on hospital care. These comparisons underscored not only technical differences but also deep emotional resonances affecting perceptions of autonomy, dignity, and quality of life:

*“(…) I started with hemodialysis, and my experience was really, really bad, very unstable at the beginning. I felt like I couldn’t take it anymore. Later on, with peritoneal dialysis, I felt more stable, less tired.”*

*—CF2*

*“My sister is on hemodialysis. Somehow, I’ve been able to keep up with my life (…) I’ve compared the benefits of peritoneal dialysis and hemodialysis (…) I think she’s really enslaved, and I would feel the same way—going to the hospital every other day.”*

*—CM4*

### 3.4. CGM as a Complementary Strategy

CGM has become a valuable tool in the management of T2DM among patients undergoing renal replacement therapy. In addition to optimizing metabolic control, its use reshaped patients’ subjective experiences of self-care and reinforced perceptions of autonomy and safety. However, technical and emotional challenges were also noted and must be considered to maximize its impact. The following subsections describe perceived benefits, limitations, and device acceptability from the patient perspective.

#### 3.4.1. Perceived Benefits: Self-Care and Prevention of Complications

CGM was perceived as an empowering tool for daily self-care. It enabled continuous visualization of glucose patterns, allowing for immediate adjustments in diet, physical activity, and insulin administration. This direct feedback not only supported better metabolic control but also reinforced motivation to sustain healthy behaviors and prevent complications:

*“Using the monitor was a good experience because I didn’t have to check all the time whenever I needed to, I could just look at the device.”*

*—CM10*

*“It helped me see how the device worked in my body over those two weeks (…) it made me more aware (…).”*

*—CF2*

#### 3.4.2. Limitations and Barriers in Clinical Practice

Despite its advantages, patients identified several technical barriers in daily CGM use. Connectivity issues, the need for frequent calibrations, and unexpected disconnections were sources of frustration and, in some cases, diminished trust in the device. These limitations may compromise adherence and, if not properly addressed, reduce the therapeutic benefits of CGM:

*“Sometimes it didn’t charge (…) there were moments when the phone got so messed up that it lost connection with the device (…)”*

*—AM3*

*“Both times I used it, it disconnected. It lasted two days, then it just stopped working (…)”*

*—AM4*

#### 3.4.3. Acceptability and Patient Recommendations

Although overall perceptions of CGM were positive, its cost was identified as a significant barrier to sustained access. Patients acknowledged the clinical and quality-of-life benefits of the device but stressed that its high price restricted availability to those who could afford it privately. Several participants proposed that CGM be integrated into institutional therapeutic strategies, either through support programs or mechanisms to improve access. From the patients’ perspective, institutional support would not only enhance equity in care but also represent a complete improvement in healthcare quality:

*“Personally, I think it’s a very, very good device—I really like it (…) I’m basically waiting for the price to go down.”*

*—HF2*

*“If I had the chance to have it or if it were provided through insurance, I’d definitely use it. Because it really works (…) For someone with diabetes, it’s amazing. Probably very expensive, but it truly works.”*

*—HF4*

Overall, the majority of participants had a positive perception of CGM. Approximately 85% noted its usefulness in facilitating self-care, increasing awareness of glycemic patterns, and reducing the need for frequent fingerstick. In contrast, about 15% voiced concerns regarding technical issues such as connectivity, frequent calibrations, and the high cost, though they did not completely reject the device. A summary of perceptions and their key arguments is provided in Table 3.

## 4. Discussion

In this qualitative study, the lived experiences of individuals with type 2 diabetes undergoing renal replacement therapy, PD or HD, were explored. The focus was placed on insulin management, use of CGM, and the emotional and social consequences of treatment. Narratives revealed clinical differences between modalities and substantial implications regarding autonomy, emotional burden, lifestyle restructuring, and economic barriers.

In Mexico, recent national data indicate that hemodialysis has become more prevalent than peritoneal dialysis, reversing the historical predominance of the latter. Despite this epidemiological shift, peritoneal dialysis, particularly automated peritoneal dialysis, remains a crucial therapeutic option due to its distinct metabolic implications, such as nocturnal exposure to glucose-based dialysate and the resulting variability in glycemic absorption. Exploring patient experiences in peritoneal dialysis is therefore essential to understand the specific challenges of insulin management and continuous glucose monitoring in this context. Hemodialysis participants, in turn, provided a contrasting perspective, particularly regarding fatigue, intradialytic hypoglycemia, and the social limitations associated with hospital-based therapy.

Participants undergoing PD reported a greater sense of glycemic control and flexibility, especially when using CGM. These observations align with prior research highlighting CGM’s role in improving self-care and reducing glycemic distress (Lawton et al., 2018 [13]; Gerogianni et al. [14]; Varela et al. [15]). Conversely, the HD experience was characterized by physical exhaustion, institutional dependence, and social restriction, aligning with findings by Wieliczko et al. [18], who documented physiological fluctuations and cumulative fatigue related to HD.

However, the present findings extend prior knowledge by highlighting the emotional significance of these treatments. While Powers et al. [19] advocated for structured educational strategies within a biomedical framework, it is suggested here that patient empowerment remains insufficient unless supported by a deeper acknowledgment of the psychosocial dimensions of chronic illness.

Insulin management in PD was perceived as uncertain due to variable glucose absorption through the peritoneal membrane, a challenge previously acknowledged by Misra and Khanna [5]. Frustration and a sense of loss of control were frequent occurrences. While intraperitoneal insulin has been suggested as an alternative, its use does not address daily fluctuations or the practical challenges faced by patients. These perceptions deserve further investigation through quantitative methods.

The perceived benefits of CGM extended beyond technical functionality. In addition to facilitating real-time therapeutic adjustments, as described by Ling et al. [7]. Emotional empowerment was also reported, attributed to the continuous feedback received from the device. Moreover, lifestyle changes required by PD dietary restrictions, limited physical activity, and social adjustments, were associated with a perceived loss of independence, confirming similar findings by Varela et al. [15]. In this context, family support emerged as a critical facilitator of emotional resilience and treatment adherence, echoing the work of Fox et al. [20] and reinforcing recent evidence that caregiver burden and psychological distress differ across dialysis modalities and transplantation settings [21].

These benefits, such as greater autonomy and a perceived improvement in safety, were reported as subjective experiences. While this is consistent with the phenomenological design of the study, future mixed-methods or quantitative research will be needed to validate their prevalence and assess their broader impact on patient care.

In interpreting these findings, it should be noted that the narratives were derived from a minimum of 14 days of CGM use, consistent with the recommendations of the American Diabetes Association (ADA), which indicate that this period provides more reliable glycemic averages. This duration ensured sufficient exposure for participants to provide informed accounts of their experiences, although it did not capture long-term adherence or sustainability. Future studies with extended follow-up will be needed to evaluate the durability of these perceptions and the feasibility of continuous CGM use in dialysis populations, particularly considering its high cost.

Therapeutic decisions, it is argued, should not rely solely on clinical outcomes but must incorporate the patient’s subjective experience. The perceived “relative freedom” of PD contrasted sharply with the “slavery” associated with frequent HD sessions, reinforcing the arguments presented by Masakane et al. [22], Kim S. et al. [23], Yu X. et al. [24], and Cassidy et al. [25], who also highlighted persistent barriers to patient education and effective shared decision-making in the chronic kidney disease population. Although CGM was positively evaluated, technical and economic limitations, such as frequent disconnections, calibration requirements, and high costs, were reported, as also noted by Ling et al. [8] and in recent reviews [12], drawing attention to inequities in access to assistive technologies.

These findings underscore the importance of integrating accessible, context-sensitive technological solutions into personalized care plans that prioritize the patient’s holistic well-being alongside glycemic control.

### Study Limitations

Certain limitations should be acknowledged. The qualitative nature of the study and the use of telephone interviews may have limited the depth of interpretation and introduced response bias. The findings are situated within the Mexican sociocultural and healthcare context, which may restrict their transferability to other settings. Diversity was enhanced by recruiting across five hospitals representing different levels of care and including patients from diverse socioeconomic contexts, which allowed us to capture a heterogeneous range of experiences. However, we did not perform subgroup analyses by socioeconomic status, and this should be addressed in future mixed-methods or quantitative studies, particularly given the importance of cost as a barrier to CGM access. Future research should also aim to quantify the perceptions reported here and examine the clinical and psychosocial effects of CGM across dialysis modalities. Additionally, interventions should be designed to encompass emotional and social dimensions, supporting a truly patient-centered model of care.

Patients identified cost as a barrier to CGM use. In Mexico, the Social Security currently provides CGM only for selected individuals with type 1 diabetes. In this study, devices were obtained exclusively for research purposes, and patient concerns about affordability emerged spontaneously during interviews. These cost-related issues were recorded as qualitative perceptions rather than formal economic outcomes. Future studies should quantify the financial implications of CGM use in dialysis populations and evaluate potential strategies for equitable access.

The main strength of this study lies in providing an in-depth exploration of patients lived experiences, offering insights that can inform patient-centered care in dialysis. Future research may complement these results with quantitative or mixed methods approaches to evaluate the prevalence and broader applicability of the identified themes.

## 5. Conclusions

The results indicate that, in addition to conventional clinical indicators, the patient’s subjective experience, including economic barriers to accessing technological innovations like CGM and the emotional challenges associated with each treatment modality, should be considered a central element in therapeutic decision-making. Including the patient’s perspective in the development of comprehensive management strategies will facilitate the creation of more humane, personalized, sustainable, and effective interventions in the joint management of diabetes and kidney disease.

## Figures and Tables

**Table 1:** Demographic data of the 50 patients with CMG in PD and HD.

Demographics Data	n		DPCA/DPA	HD
Age (year) $\overset{¯}{x}$ ± SD	50	59.14 ± 10.10	58.8 ± 10.33	57.0 ± 9.99
Gender	50	f (%)		
Female		23 (46)	16 (30)	7 (70)
Male		27 (54)	24 (60)	3 (30)
Educational level	50		40	10
No academic instruction		4 (8)	3 (8)	1 (10)
Primary School		19 (38)	13 (33)	6 (60)
Middle school		9 (18)	7 (16)	2 (20)
High School		15 (30)	15 (38)	
Professional		3 (6)	2 (5)	1 (10)
Marital Status	45		38	7
Single		10 (21)	8 (21)	2 (29)
Married		37 (79)	30 (79)	5 (71)
Self sufficient	40		31	9
Yes		20 (50)	12	8 (89)
No		20 (50)	19 (61)	1 (11)
		$\overset{¯}{x}$ ± SD	$\overset{¯}{x}$ ± SD	$\overset{¯}{x}$ ± SD
Weight (kg)	47	70.7 ± 16.32	70 ± 17.10	67 ± 10.33
Height (cm)	46	160.5± 10.28	162.5 ± 10.97	160.5 ± 10.05
IMC	39	27.1 ± 4.78	27.5 ± 4.58	24.8 ± 4.16
Heart Rate	43	79.5 ± 9.51	80 ± 9.51	76 ± 10.50
BP Systolic	43	137.9 ± 20.85	136 ± 21.96	145 ± 19.29
BP Diastolic	41	80.8 ± 12.91	80 ± 13.40	88 ± 12.46

IMC: Body Mass Index (BMI), calculated as weight in kilograms divided by height in meters squared (kg/m^2^). BP Systolic: Systolic Blood Pressure, measured in millimeters of mercury (mmHg). BP Diastolic: Diastolic Blood Pressure, measured in millimeters of mercury (mmHg). Note: The total sample comprised 50 participants; missing values in some variables (e.g., weight, height, BMI, and blood pressure) reflect incomplete records.

**Table 3:** Patients’ perceptions on the use of GCM.

Posture	Approximate Percentage	Short Explanation	Basis
Agreement	85%	Most patients value the CGM for allowing less invasive monitoring, increasing their awareness of glycemic patterns, facilitating dietary adjustments, reducing the need for frequent punctures, and improving their sense of security and self-care.	Analysis of favorable speeches of patients: CM10, CF2, HF2, CM4, CF1, AM1, HF4, CM2, AM2, HF1, CF3, CM6, AM6, CF4, AM5, HM1, AM3, AM5, CF6, HF5, HF3, AM8, CM9, HF6, AM4, CF5, CM8, AM7, CF7, HF7, CM5, CM7, AM9, HF8, CF8, CM1, AM10, CF9, HF9, HF10.
Opposed or with reservations	15%	A minority group expressed limitations related mainly to technical problems (disconnections, calibrations) and the high cost, which generated frustration and anxiety in some cases, although without totally rejecting the tool.	Analysis of critical patient speeches: AM3, AM4, HM1, HF4, CM9, CF3, HF2.

**Table 2:** Follow-up topics and subtopics.

Theme	Subtopics
Glucose management in peritoneal dialysis patients	Insulin Use in Peritoneal Dialysis Impact of continuous glucose monitoring Perception of Safety and Metabolic Control
Lifestyle changes and adaptation in peritoneal dialysis	Adjustments in diet and physical activity Transformations in social and emotional life Family support as a key element for self-care
Hemodialysis treatment experience	Physical and emotional perception of hemodialysis. Impact on work and daily life Subjective comparisons between hemodialysis and peritoneal dialysis
Continuous Glucose Monitoring as a Complementary Strategy	Perceived benefits: self-care and prevention of complications. Limitations and barriers of CGM in clinical practice Acceptability and Patient Recommendations

## Data Availability

The data supporting this qualitative study’s findings are not publicly accessible due to privacy and ethical concerns. The interview transcripts include personally sensitive information from patients with chronic kidney disease and diabetes, and sharing them publicly would breach confidentiality agreements approved by the institutional ethics committee. Reasonable requests for anonymized data can be considered by the corresponding author and the ethics board, pending institutional approval.

## Abbreviations

The following abbreviations are used in this manuscript:

CGM	Continuous Glucose Monitoring
CKD	Chronic Kidney Disease
DM2	Type 2 Diabetes Mellitus
PD	Peritoneal Dialysis
HD	Hemodialysis
ESRD	End-Stage Renal Disease
HbA1c	Glycated Hemoglobin
QOL	Quality of Life
IRB	Institutional Review Board
SDM	Shared Decision-Making
